# Structural Basis of Tau Interaction With BIN1 and Regulation by Tau Phosphorylation

**DOI:** 10.3389/fnmol.2018.00421

**Published:** 2018-11-14

**Authors:** Alessia Lasorsa, Idir Malki, François-Xavier Cantrelle, Hamida Merzougui, Emmanuelle Boll, Jean-Charles Lambert, Isabelle Landrieu

**Affiliations:** ^1^CNRS UMR8576, Lille University, Lille, France; ^2^INSERM UMR1167, Pasteur Institute Lille, Lille University, Lille, France

**Keywords:** protein-protein interaction, nuclear magnetic resonance spectroscopy, Alzheimer’s disease, bridging integrator-1 BIN1, SH3 domain, tau, phosphorylation

## Abstract

Bridging integrator-1 (*BIN1*) gene is associated with an increased risk to develop Alzheimer’s disease, a tauopathy characterized by intra-neuronal accumulation of phosphorylated Tau protein as paired helical filaments. Direct interaction of BIN1 and Tau proteins was demonstrated to be mediated through BIN1 SH3 C-terminal domain and Tau (210–240) peptide within Tau proline-rich domain. We previously showed that BIN1 SH3 interaction with Tau is decreased by phosphorylation within Tau proline-rich domain, of at least T231. In addition, the BIN1/Tau interaction is characterized by a dynamic equilibrium between a closed and open conformations of BIN1 isoform 1, involving an intramolecular interaction with its C-terminal BIN1 SH3 domain. However, the role of the BIN1/Tau interaction, and its potential dysregulation in Alzheimer’s disease, is not yet fully understood. Here we showed that within Tau (210–240) peptide, among the two proline-rich motifs potentially recognized by SH3 domains, only motif P^216^TPPTR^221^ is bound by BIN1 SH3. A structural model of the complex between BIN1 SH3 and Tau peptide (213–229), based on nuclear magnetic resonance spectroscopy data, revealed the molecular detail of the interaction. P216 and P219 within the proline-rich motif were in direct contact with the aromatic F588 and W562 of the BIN1 SH3 domain. The contact surface is extended through electrostatic interactions between the positively charged R221 and K224 residues of Tau peptide and those negatively charged of BIN1 SH3, corresponding to E556 and E557. We next investigated the impact of multiple Tau phosphorylations within Tau (210–240) on its interaction with BIN1 isoform 1. Tau (210–240) phosphorylated at four different sites (T212, T217, T231, and S235), contrary to unphosphorylated Tau, was unable to compete with the intramolecular interaction of BIN1 SH3 domain with its CLAP domain. In accordance, the affinity of BIN1 SH3 for phosphorylated Tau (210–240) peptide was reduced, with a five-fold increase in the dissociation constant, from a Kd of 44 to 256 μM. This study highlights the complexity of the regulation of BIN1 isoform 1 with Tau. As abnormal phosphorylation of Tau is linked to the pathology development, this regulation by phosphorylation might have important functional consequences.

## Introduction

Alzheimer’s disease (AD) is the most common form of dementia and the most prevalent tauopathy. In genome-wide association studies, variants of the *BIN1* gene coding for bridging integrator-1 (also known as amphiphysin 2) have been identified as the second highest genetic risk factor for AD after the *ApoE* gene, (Seshadri et al., 2010; Genin et al., 2011; Lambert et al., 2013). Several hypothesis have emerged on how BIN1 protein contributes to the increased risk of developing AD, which could involve both Aβ and Tau neurodegeneration pathways (Chapuis et al., 2013; Calafate et al., 2016; Ubelmann et al., 2017). The *BIN1* gene is subject to extensive tissue-specific alternative splicing, leading to several isoforms with different cellular localizations and functions. BIN1 protein isoforms 1–7 are expressed in the brain, isoform 8 is expressed in the muscle, and isoforms 9 and 10 are expressed ubiquitously (Prokic et al., 2014). All BIN1 isoforms contain a N-terminal Bin/amphiphysin/Rvs (BAR) domain and a C-terminal SH3 domain (BIN1 SH3) (Prokic et al., 2014). The BAR domain has a role in membrane remodeling by inducing curvature (Frost et al., 2009), and the SH3 domain mediates BIN1’s interactions with several partners (such as dynamin). However, only the brain-specific BIN1 isoforms contain a clathrin and AP-2-binding domain (CLAP) that mediates the interaction with clathrin and AP2 – suggesting a specific role for BIN1 in clathrin-mediated endocytosis in the brain (David et al., 1996; Takei et al., 1999). BIN1 was recently shown to negatively regulate this process and it has been proposed that lower Bin1 expression may favor Tau propagation through increased endocytosis (Calafate et al., 2016). Regulation of endosomal trafficking by BIN1 is also proposed to affect Aβ42 endocytic production by negatively regulating APP and BACE1 convergence in early endosomes (Ubelmann et al., 2017). BIN1 loss of function thus prevents APP and BACE1 segregation at early endosomes and may boost Aβ accumulation.

The *BIN1* gene is the first of the sporadic AD genetic determinants to have been linked to a Tau pathology (Chapuis et al., 2013). *BIN1* is upregulated in the AD brain, and its expression correlates with the neurofibrillary tangle pathology caused by intraneuronal aggregates of abnormally phosphorylated Tau proteins (Chapuis et al., 2013; Holler et al., 2014). Furthermore, the *BIN1* homolog *AMPH* mediates Tau toxicity in a *Drosophila melanogaster* model overexpressing Tau (Chapuis et al., 2013; Dourlen et al., 2017). BIN1 directly interacts with Tau and the BIN1/Tau complex is observed at specific locations in primary neurons (partly co-localized with the actin cytoskeleton network) (Sottejeau et al., 2015). BIN1 was indeed shown to modulate actin dynamics by both having actin bundling activity and stabilizing Tau-induced actin bundles (Dräger et al., 2017). Deregulation of BIN1/Tau complex formation may thus impact neuronal functions.

During the disease progression, Tau presents both an increased phosphorylation level and a modification of its pattern of phosphorylation. Tau is phosphorylated by various kinases, including proline-directed kinases [like CDK5 (Baumann et al., 1993; Kimura et al., 2014), GSK3 (Ishiguro et al., 1993; Cho and Johnson, 2003), and ERK2 (Drewes et al., 1992)], that mainly target the proline rich domain (PRD) of Tau (Landrieu et al., 2010; Leroy et al., 2010; Qi et al., 2016). For example, the AT8 diagnostic monoclonal antibody [pS202 + pT205 + (pS208) epitope] can be used to track the spatial progression of the pathology in the AD brain over time (Braak et al., 2006). Given that Tau has so many phosphorylation sites, characterizing the impact of these modifications on Tau functions is an analytical challenge (Lippens et al., 2012a,b). Yet, it remains of interest to decipher the functional consequences of specific Tau phosphorylation, and in the case of the present study, how it could modulate the BIN1/Tau interaction.

We have previously reported that the BIN1 SH3 binds directly to Tau PRD (Sottejeau et al., 2015). The interaction motif within the Tau PRD was narrowed down to a sequence located between amino acid residues 210–240, which contains two PXXP SH3 binding motifs (Sottejeau et al., 2015). This interaction is weakened by phosphorylation of the Tau PRD (Sottejeau et al., 2015), and in particular, phosphorylation of T231 (Sottejeau et al., 2015). Furthermore, we recently showed that the SH3 domain of the neuronal-specific BIN1 isoform 1 protein is engaged in a dynamic equilibrium between open and closed conformations (Malki et al., 2017). The closed conformation results from BIN1-SH3’s interaction with the BIN1-CLAP domain. Tau (210–240) is able to shift the equilibrium toward the intermolecular interaction – indicating a subtle regulation of the Tau/BIN1 interaction.

As studying the regulation of the BIN1/Tau interaction is an important step in understanding its specific contribution to the AD process, we further pursued this investigation. Here, we first determined which PXXP motif(s) is(are) critical for the BIN1/Tau interaction. Based on the NMR data, we proposed next a structural model of the complex between BIN1 SH3 and a Tau peptide, which provides the atomic detail of the interaction. Lastly, we looked at whether Tau (210–240) phosphorylation affects the dynamic equilibrium between open and closed conformations of BIN1 isoform 1.

## Materials and Methods

### Proteins and Peptides

The BIN1 isoform 1 and BIN1 SH3 domain were expressed and purified as previously described (Malki et al., 2017). cDNA encoding Tau (210–240) peptide SRTPSLPTPPTREPKKVAVVRTPPKSPSSAK was cloned into the pETNKI-HisSUMO3-LIC-Kan vector following a ligation-independent cloning procedure (Luna-Vargas et al., 2011). Uniformly labeled ^15^N and ^13^C or ^15^N protein/peptide samples were produced in M9 medium supplemented with 0.1 mM CaCl_2_ and 2 mM MgSO_4_ and containing 0.1% ^15^NH_4_Cl and 0.2% ^13^C-glucose (Sigma-Aldrich) or 0.4% glucose, with 0.5 g/l of ^15^N- and ^13^C-enriched or ^15^N-enriched ISOGROW (Sigma-Aldrich), depending of the labeling scheme. The recombinant His_6_-SUMO Tau (210–240) was purified using a nickel affinity chromatography column and then incubated 16 h with SENP2 protease in the presence of 2 mM DTT, at 4°C. The protein sample was buffer-exchanged using a desalting column (G25 resin, cutoff of 7 kDa; PD-10 GE Healthcare) against 50 mM sodium phosphate pH 7.3, 30 mM NaCl, 3 mM DTT, and then loaded on a nickel affinity chromatography column. The tag-cleaved peptide was then recovered in the flow-through.

### NMR Spectroscopy

NMR experiments were recorded on Bruker 950-MHz, 900-MHz, or 600-MHz spectrometers all equipped with a triple-resonance cryogenic probe. NMR measurements were performed in 50 mM sodium phosphate buffer, pH 7.3, 30 mM NaCl, 3 mM DTT and 10% D_2_O (NMR buffer). BIN1 SH3 domain backbone assignment has been previously reported (Malki et al., 2017). Tau (210–240) backbone assignment (0.5 mM sample) was performed at 600 MHz by recording 3D HNCA, 3D HN(CO)CA, 3D H(CA)NH, 3D HNCO, 3D HN(CA)CO 3D CBCA(CO)NH, and 3D CBCANH. For side-chain assignment, additional 3D^15^N NOESY-HSQC, 3D^15^N-edited TOCSY, 3D (H)CCH TOCSY, and 3D H(C)CH TOCSY experiments were recorded. A 3D ^13^C-edited NOESY was further used for side-chain assignment validation. All assignment experiments were recorded at 5°C. Chemical shift values (Supplementary Table S1) were directly transferred at 20°C, because the differences of chemical shift values were small between 5 and 20°C. An analogous set of experiments was recorded for phosphorylated Tau (210–240) backbone assignment (Supplementary Table S2). The H^α^C^α^N spectra (Wang et al., 1995) of Tau (210–240) peptide at 0.5 mM were recorded at 20°C with the standard Bruker pulse sequence in the NMR buffer containing 100% D_2_O. BIN1 isoform 1 ^1^H-^15^N HSQC spectra were recorded at 20°C at 900 MHz (0.08 mM sample) with 3096 points (direct) and 280 points (indirect), for spectral width of 14 ppm and 26 ppm, respectively, and 256 scans. A 3D F1 ^13^C/^15^N-filtered and F3 ^15^N-edited NOESY-HSQC, with WATERGATE (WATER suppression by GrAdient-Tailored Excitation) scheme (Piotto et al., 1992), named noesyhsqcf3gpwgx13d, part of the Bruker pulse sequence library, was recorded at 20°C at 950 MHz for the detection of intermolecular NOEs to amide groups. This data was used to solve the structure of the complex between BIN1 SH3 domain and Tau (210–240). Spectra were processed using TopSpin software (Bruker) and analyzed by CcpNmr Analysis (Vranken et al., 2005).

### ^15^N Relaxation NMR Experiments

^15^N-R1, ^15^N-R2, and {^1^H}-^15^N steady-state heteronuclear NOE spectra were recorded at 293K on ^15^N-labeled samples (0.12 mM) at 600 MHz for BIN1 SH3 and BIN1 SH3/Tau (210–240) complex (1:8 molar ratio). For the R1 experiments (Supplementary Figure S1), 11 data points were recorded, using relaxation delays between 50 and 1300 ms and a recycle delay of 4 s. For R2 experiments (Supplementary Figure S1), 13 data points were recorded, using relaxation delays between 10 and 150 ms (recycle delay was 4 s). The heteronuclear NOE experiment (Supplementary Figure S1) was recorded by including, or not, a 4 s period of 120° ^1^H saturation pulses, separated by 5 ms. Recovery delay was 4 s. Uncertainties in peak intensities were estimated from one triplicate data point.

### HADDOCK Docking

The HADDOCK 2.2 (Dominguez et al., 2003; van Zundert et al., 2016) program was used to calculate the structure of the BIN1 SH3/Tau peptide complex, based on experimental unambiguous and ambiguous restraints. The NMR structure of BIN1 SH3 extracted from a BIN1 SH3 complex (PDB 5i22) (Tossavainen et al., 2016) was used as a starting point for the docking. In addition, a structural model of Tau (213–229) peptide was built, using the Coot program (Emsley and Cowtan, 2004), starting from the Chikungunya virus nsP3 peptide (1728–1744) in interaction with BIN1 SH3 (PDB code 5i22). The docking was next driven under defined intermolecular unambiguous restraints from a NOESY spectrum and ambiguous restraints chosen as the residues involved in intermolecular NOEs. Intermolecular NOEs were identified between BIN1 SH3 residues 557, 558, 561, 587, and 588, and Tau (213–229) residues 228/229, 226, 223, 219, and 216, respectively (Supplementary Figure S2 and Supplementary Table S3). The docking algorithm includes three consecutive steps: (i) rigid body energy minimization (1000 generated structures) (ii) semi-flexible simulated annealing of the 200 lowest energy structures from step (i) and (iii) flexible explicit solvent refinement (200 structures, 8.0 -Å shell of TIP3P water molecules). All calculations were run through the WeNMR Web portal using the expert interface. A total of 196 final models were grouped into two clusters based on their interface-ligand RMSD, using a cutoff of 5 Å. The final overall score of each cluster (Supplementary Table S4) is based on the four highest HADDOCK scores of the models in that cluster. The best cluster, cluster 1, contained 190 structures (Supplementary Table S4), z score = −1.0). The molecular structure of the complex was viewed and represented using CHIMERA.

### Phosphorylation of Tau (210–240)

Phosphorylation of SUMO-Tau (210–240) was obtained by incubation with recombinant CDK2/CycA3 kinase (Welburn and Endicott, 2005) (molar ratio 1/100), for 3 h at 37°C, in the presence of 2 mM ATP, 2.5 mM MgCl_2_, 2 mM DTT, and protease inhibitors in 50 mM HEPES, pH 8.0. The incubation was repeated once after exchange into fresh buffer and upon new addition of kinase. The His-SUMO tag was removed after phosphorylation, as described here above for the unmodified peptide. Completion of phosphorylation was assessed by NMR 2D ^1^H-^15^N HSQC of the ^15^N-Tau (210–240) peptide and MALDI-TOF (Matrix Assisted Laser Desorption Ionization – Time of Flight) mass spectrometry. Mass analysis was performed in ion-positive reflector mode on an ABI Voyager DE-Pro MALDI-TOF mass spectrometer (Applied Biosystems), using as matrix a saturated solution of α-cyano-4-hydroxycinnamic acid in CH_3_CN:H_2_O:CF_3_COOH (50:50:0.1). Phosphorylated Tau (210–240) ^1^H-^15^N HSQC spectrum was recorded at 600 MHz, peptide concentration 0.5 mM, resolution 2048 points in the direct dimension and 256 points in the indirect dimension, spectral width 14 and 17 ppm, respectively, and 16 scans. Based on the relative intensities of the NMR resonance signals (Supplementary Figure S3), full modification was observed for residues T212, T231, and S235 while residue T217 was half-phosphorylated. Results from MS analysis were in agreement (Supplementary Figure S4). All the purification steps were performed at 4°C in the presence of a protease inhibitor cocktail (Sigma-Aldrich).

### Dissociation Constant Determination

Titration of 70 μM ^15^N-BIN1 SH3 with increasing amount of unlabeled Tau (210–240) (from 35 to 840 μM) peptides was performed, monitoring the complex formation by the gradual ^1^H and^15^N chemical shift change of the resonances in ^1^H,^15^N HSQC, at pH 7.3 (50 mM phosphate buffer) and 20°C. The weighted average chemical shift differences were calculated as described by Garrett et al. (1997) - i.e., (ΔH^2^ + (ΔN×0.2)^2^)^1/2^, with ΔH and ΔN the chemical shift changes for ^1^H and ^15^N, respectively. Dissociation constants were obtained by fitting the chemical shift perturbation data to the following equation Δδobs = Δδmax(a + b + K_d_−((a + b + K_d_)^2^−4ab)^1/2^)/2a where Δδobs is the weighted average of the chemical shifts in the free and bound states and Δδmax is the maximal signal change upon saturation. K_d_ is the dissociation constant, *a* and *b* are the total peptide and BIN1 SH3 concentrations, respectively. K_d_ were averaged based on chemical shift perturbations of 7 distinct resonances.

## Results

### Proline Assignment in Tau (210–240) Peptide

**FIGURE 1:**
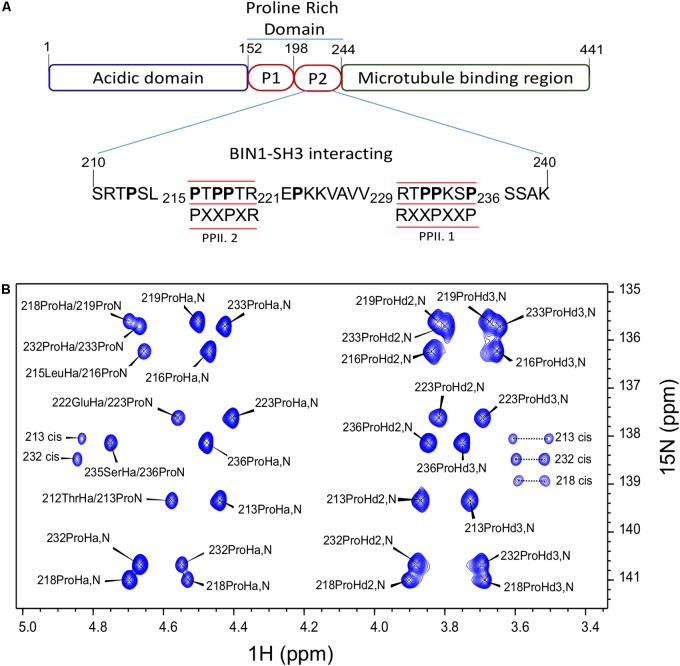
Assignment of Tau (210–240) proline residues. **(A)** Domain composition of the largest isoform of the neuronal Tau protein. P1 and P2 are the proline-rich regions. Tau (210–240) sequence is shown as part of the PRD. The proline residues are indicated in bold characters. The canonical SH3 class I and II binding motifs are underlined and the consensus motifs are given below. PPII.1 and PPII.2 are PPII helix motif class I and II, respectively. **(B)** Assigned 2D HA(CA)N spectrum of the prolines in Tau (210–240) peptide at 20°C.

We have previously shown direct interaction of the BIN1 SH3 (BIN1 SH3) with the PRD of Tau [PRD, Tau (165–245)] (Sottejeau et al., 2015). The interaction motif within Tau PRD was narrowed-down to a sequence located between amino acid residues 210–240. In this previous work, ^1^H-^15^N HSQC spectra were used for chemical shift perturbation analysis. As ^1^H-^15^N HSQC experiment correlates the resonances of directly bound ^1^H and ^15^N nuclei, the majority of the amino acid residues in the peptide was represented in this analysis, except proline residues, due to the lack of the backbone amide proton. However, Tau (210–240) has eight proline residues organized in two different canonical SH3 binding sites (Feng et al., 1994; Lim et al., 1994). One of class I, PPII.1, between residues 230 to 236, and another one of class II, PPII.2, between amino acid residues 216 and 221 (Figure 1A). To investigate the role of these proline residues in Tau (210–240)/BIN1 SH3 interaction, a specific NMR strategy was used, based on H^α^-^15^N correlation, allowing sequential assignment of molecules rich in prolines (Kanelis et al., 2000; Ahuja et al., 2016). All the proline residues in Tau (210–240) were assigned, including their minor *cis* conformers (Figure 1B).

### Tau (210–240) Fragment Interacts With BIN1 SH3 Domain Using PPII.2 Motif

**FIGURE 2:**
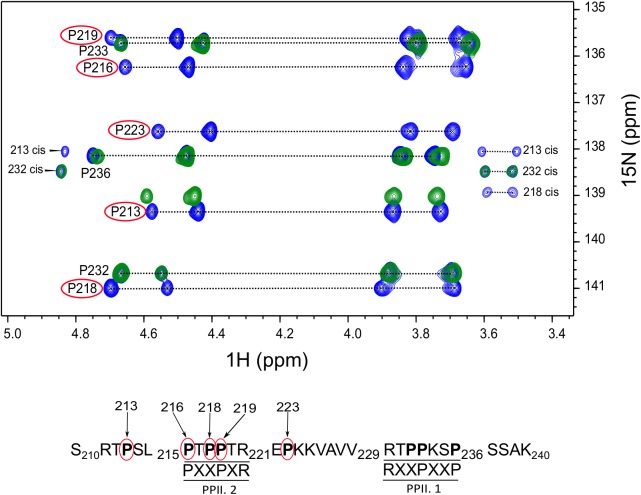
Critical proline residues involved in Tau binding to BIN1. The 2D HA(CA)N spectrum of Tau (210–240) peptide free or in the presence of BIN1 SH3 domain molar ratio 1:4 is shown in blue and green, respectively. The proline residues affected by the presence of BIN1 SH3 domain are indicated by red circles. In addition, the position of each affected proline residue within Tau (210–240) sequence is numbered in the sequence below the spectrum.

After assignment of all the proline residues in Tau (210–240) fragment, we next looked at whether the two proline rich motifs of Tau (210–240) were involved in the interaction with BIN1 SH3 domain. Comparison of the 2D HA(CA)N spectra of Tau (210–240) in the absence and presence of an excess of BIN1 SH3 domain showed that all resonances corresponding to proline residues in the proline-rich motif PPII.2 disappeared in the presence of the BIN1 SH3 domain (Figure 2). Similarly, peaks corresponding to P223 at the C-terminal end of the PPII.2 were broadened beyond detection, whereas those of P213, in the N-terminal of PPII.2, were less affected and only showed minor chemical shift modifications. Resonances of proline residues in the proline-rich motif PPII.1 were barely affected, neither by broadening nor by chemical shift modification. This result clearly indicated that Tau (210–240) interacts with BIN1 SH3 domain using the PPII.2 proline-rich motif within its sequence.

### Structural Model of the BIN1 SH3/Tau (210–240) Peptide Complex

**FIGURE 3:**
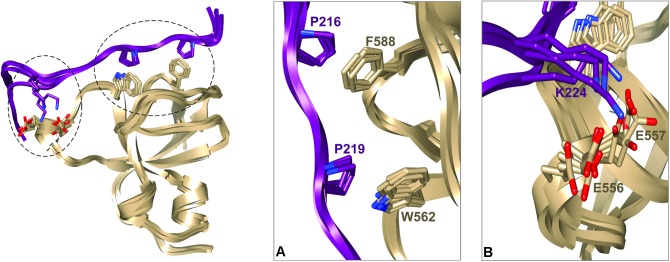
Representative structural models of BIN1 SH3/Tau (213–229) complex corresponding to the four best structures of HADDOCK calculation from cluster 1 (**A).** Ribbon representation of BIN1 SH3 (in gold)/Tau (213–229) (in violet). Side-chains of the main residues involved in the interaction are represented as sticks **(B)**. Enlarged views of the complexes corresponding to the encircled regions in **(A)**.

**FIGURE 4:**
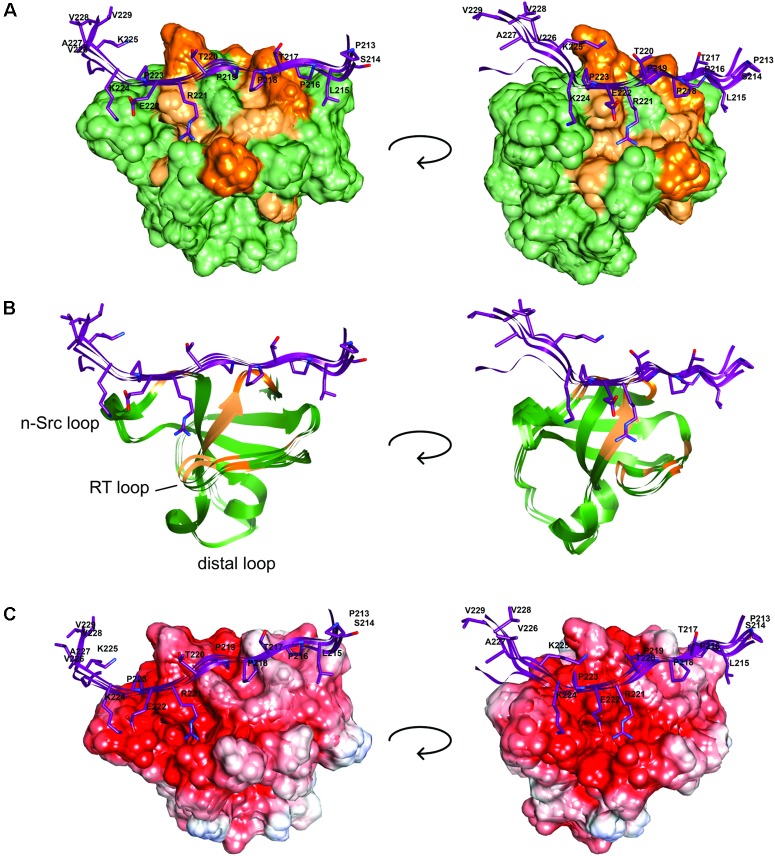
BIN1 SH3 interaction with Tau (213–229) **(A)**. The solvent accessible molecular surface of BIN1 SH3 (in green) is highlighted with the major ^1^H,^15^N combined chemical shift perturbations observed upon addition of Tau (210–240) peptide to BIN1 SH3, dark orange > 0.3 ppm, light orange > 0.2 ppm (as in Malki et al., 2017, see Figure 6). **(B)** Same as A., in ribbon representation. **(C)** The electrostatic potential is colored on the molecular surface of BIN1 SH3 from negative values in red (-8 keV) to positive values in blue (+5 keV). Tau (213–229) is represented as ribbon on the surface (in violet), with the side-chains of Tau peptide residues shown as sticks.

We next used these data as a starting point to obtain a structural model of BIN1 SH3 in complex with Tau (210–240) peptide. First, NMR dynamics experiments provided insights into the complex formation. ^15^N-relaxation measurements of ^15^N BIN1 SH3 showed homogenous R1 and R2 values, compatible with a compact structure (Supplementary Figure S1). A correlation time τc of 6.3 ± 0.3 ns was calculated, based on the average R1 and R2 values, as expected for a monomeric BIN1 SH3 domain in the NMR samples (Supplementary Figure S1). In the presence of the peptide, average R1 values were increased while average R2 values were decreased, corresponding to an increase in the molecular weight of the system. A correlation time τc of 9.7+/−0.4 ns was calculated for BIN1 SH3/Tau (210–240) complex, based on the average R1 and R2 values, compatible with a monomeric state of a 1:1 complex. NMR data were next used to build a model of the complex, using HADDOCK program. The docking was started from an NMR structure of BIN SH3 (Tossavainen et al., 2016) and a model of Tau (213–229) peptide. Starting from these two initial individual structures, the docking was conducted under restraints derived from the NMR experimental data (Supplementary Figure S2 and Supplementary Table S3). The four best structures resulting from the docking calculation, in the cluster of conformations showing the best statistics (cluster 1 in Supplementary Table S4), were chosen as being representative of BIN1 SH3/Tau (213–229) complex (Figures 3, 4). BIN1 SH3 accommodated the P^216^xxP^219^ consensus motif of Tau peptide (Figures 1, 2) into the canonical hydrophobic xP binding pocket of the SH3 domain (Figure 3). Specific hydrophobic interactions were observed between the side-chains of F588 on the BIN1 SH3 side and P216 on the Tau peptide, and between the side-chains of W562 and P219 (Figure 3A). In contrast, P218 side-chain pointed to the opposite direction with respect to P219 and was not interacting with BIN1 SH3 domain. Moreover, residues E556 and E557 in the n-Src loop of the specificity zone of BIN1 SH3 provided an additional anchoring point. Both E556 and E557 side-chains were oriented toward the Tau (213–229) peptide, in close proximity to K224 side-chain (Figure 3B). Mutation of K224 and K225 into alanines in Tau (210–240) peptide indeed resulted in a reduced binding affinity to the BIN1-SH3 domain, with a dissociation constant K_d_ of 44 ± 3 μM for Tau (210–240) peptide increasing by a factor of ten to 429 ± 94 μM for the mutated peptide (Supplementary Figure S3). Finally, at the C-terminus of the docked Tau (213–229) peptide, V228/V229 H_α_ protons were also in close contact with E557 backbone H-N, as observed by intermolecular NOEs (Supplementary Table S3). The position of Tau peptide on the surface of BIN1 SH3 showed a good match with the major chemical shift perturbations of BIN1 SH3 ^1^H, ^15^N resonances on addition of Tau (210–240) peptide (Figures 4A,B), highlighting the β5 strand, the n-Src loop and in addition the RT loop (Figure 4B). Reporting the electrostatic potential on the BIN1 SH3 surface additionally showed positioning of the R221 and K224 side-chain on a negatively charged region (Figure 4C). The structure of the BIN1 SH3/Tau (213–229) peptide complex showed involvement in the binding of both the xP pocket and the specificity region of BIN1 SH3 (n-Src loop), mediated on the Tau side mainly by P216/P219 and R221/K224, respectively (Figure 4).

### Interaction of BIN1 Isoform 1 With Tau (210–240) Is Weakened by Phosphorylation

**FIGURE 5:**
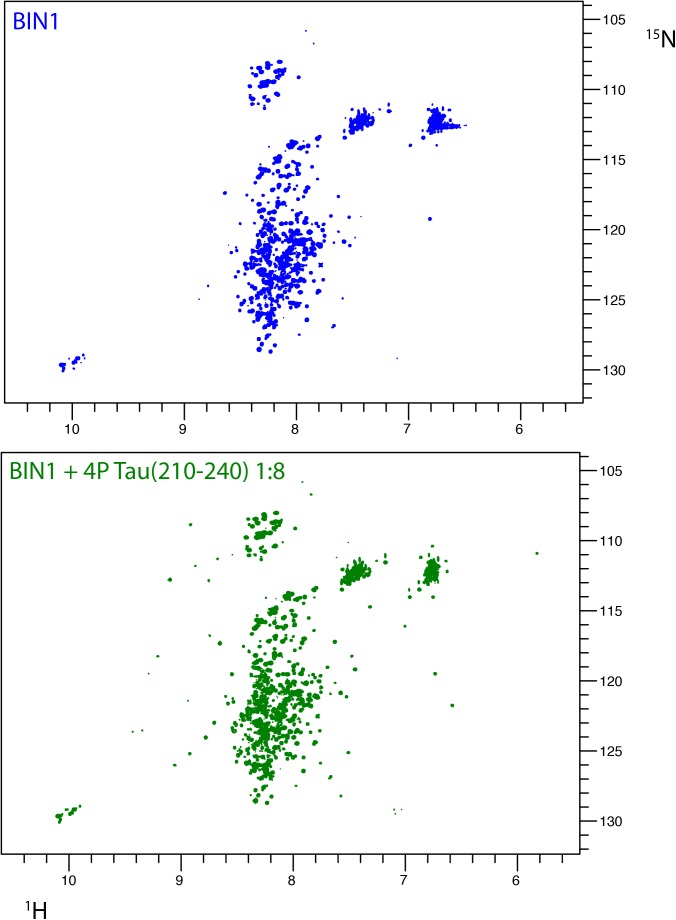
Interaction of BIN1 isoform 1 with Tau (210–240) and impact of multiple Tau phosphorylation on the interaction. **(A)** ^1^H-^15^N HSQC spectrum of BIN1 isoform 1 (112 KDa, homodimer). Because of the large molecular weight of BIN1, which lead to severe line broadening, its spectrum displayed, mostly in its central part, signal of low spectral dispersion, typical of disordered regions of the protein. **(B)** ^1^H-^15^N HSQC spectra of BIN1 isoform 1 in the presence of phospho-Tau (210–240), molar ratio 1:8. The signals corresponding to the isolated BIN1 SH3 domain were barely detected. Phospho-Tau (210–240) cannot compete with the intramolecular interaction of BIN1 SH3 with the CLAP domain.

**FIGURE 6:**
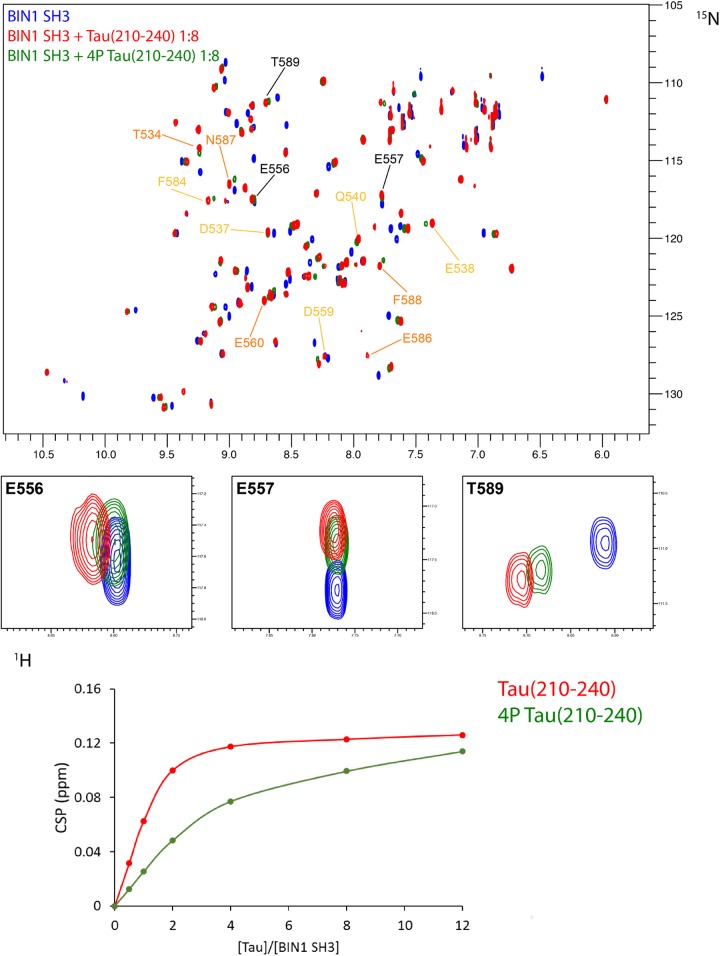
Affinity of BIN1 SH3 for Tau (210–240), phosphorylated, or not. **(A)** Superimposed 2D ^1^H-^15^N HSQC spectra of 70 μM ^15^N BIN1 SH3, free in solution (in blue) and with 8 molar amount of Tau (210–240) (overlaid in red) or phosphorylated Tau (210–240) (overlaid in green). Labeled resonances correspond to the major chemical shift perturbations, color coded as in Figure 4A. Enlarged regions of the superimposed spectra show 3 resonances involved in the interaction. **(B)** Gradual chemical shift perturbation of residue D537 ^1^H, ^15^N resonance of BIN1 SH3 (70 μM) *versus* increasing amount of Tau (210–240) (red) or phosphorylated Tau (210–240) (green).

We previously showed that BIN1 SH3 domain was engaged in an intramolecular interaction with BIN1 isoform 1 own CLAP domain (Malki et al., 2017). Tau (210–240) peptide was able to compete with this interaction, shifting the dynamic equilibrium from a closed to an open conformation. As NMR is sensitive to mobility, this equilibrium translated into detection of BIN1 SH3 domain signals only upon its release from the intramolecular interaction (Malki et al., 2017). We further addressed whether such equilibrium between open and closed states of BIN1 isoform 1 might be affected by phosphorylation of Tau (210–240). A ^1^H-^15^N HSQC spectrum of BIN1 isoform1 was thus acquired in the absence or presence of an excess of Tau (210–240) phosphorylated at residues T212, T217, T231, and S235 (Supplementary Figures S4), all proline-directed sites within this peptide. Addition of phosphorylated Tau (210–240) peptide to BIN1 isoform1 sample did not result in the same changes in the spectrum (Figure 5, compare A with B), although some weak signals from BIN1 SH3 were still visible. This indicated that Tau (210–240) phosphorylated at multiple sites was not able to compete with the intra-molecular interaction of BIN1 SH3 with the CLAP domain as efficiently as its unphosphorylated counterpart. Indeed, comparison of the affinity of the isolated BIN1 SH3 domain for the Tau peptide (210–240), unphosphorylated or not, showed an increase of the dissociation constant K_d_, from 44 ± 3 μM to 256 ± 12 μM for the phosphorylated peptide (Figure 6 and Supplementary Figure S5). Consequently, in the presence of the phosphorylated Tau peptide (210–240), BIN1 SH3 domain remained immobilized by the intramolecular interaction with BIN1 isoform1 CLAP domain, mainly adopting the closed conformation.

## Discussion

*BIN1* gene was found as a genetic determinant of AD, allowing to address new patho-physiological hypothesis (Lambert et al., 2009; Seshadri et al., 2010). A first step in deciphering the role of BIN1 in AD dysfunctions was taken by the discovery of its direct interaction with Tau protein (Chapuis et al., 2013), a well-known actor of the disease. The specific function of the interaction of BIN1 with Tau, however, remained to date to be uncovered, and in this context emphasized the need to grasp its molecular detail. Here we first sought the atomic detail of the interaction of BIN1 SH3 with Tau. The BIN1 interaction motif within Tau PRD is a sequence located between amino acid residues 210–240, containing two potential SH3-binding motifs (Sottejeau et al., 2015; Malki et al., 2017). The binding site for the BIN1 SH3 within this sequence was found here to be specific for a canonical SH3 class II binding motif PXXPX+ (Feng et al., 1994; Lim et al., 1994) corresponding to P_216_TPPTR_221_. The identification was based on HA(CA)N spectra allowing for the detection of the resonances specific for the proline residues, which are absent in a classical 2D ^1^H,^15^N HSQC spectrum. Assignment of the Pro residues matched previous assignment of Pro residue resonances in peptides and fragments of Tau encompassing Tau (210–240) (Smet et al., 2004, 2005; Ahuja et al., 2016). Our analysis also confirmed that the proline residues mainly adopt the *trans* conformation (Smet et al., 2004, 2005; Ahuja et al., 2016). The BIN1 SH3/Tau (213-229) peptide complex structure further showed that the consensus motif is bound to the hydrophobic xP binding pocket of BIN1 SH3, in which P216 and P219 are in direct contact with the aromatic F588 and W562 residues on the domain side. The contact surface is increased through electrostatic interactions between the positively charged residues on the Tau peptide side, namely R221 and K224, and those negatively charged on the protein side within the n-Src loop, corresponding to E556 and E557. These negatively charged residues, located in the so-called specificity region of BIN SH3 (Pineda-Lucena et al., 2005), explain the importance of basic residues in the binding affinity. Further interactions involved residues V228 and V229 of Tau peptide, which appeared to be oriented toward the protein.

We have previously showed that BIN1 interaction with Tau is decreased by phosphorylation of Tau, of at least T231 (Sottejeau et al., 2015). In addition, a competition for BIN1 SH3 domain binding to Tau was shown by BIN1 Isoform 1 own CLAP domain, in an intramolecular mechanism of regulation (Malki et al., 2017). Addition of an excess of Tau to BIN1 is able to displace the intramolecular interaction, in profit of an intermolecular interaction with Tau proline-rich motif (Malki et al., 2017). These results demonstrated the complex regulation of the BIN1/Tau interaction and the need to understand all the key elements of this regulation mechanism. Here, we looked at both aspects of the regulation of this interaction: how phosphorylation of Tau (210–240) peptide on sites surrounding the proline-rich SH3-binding consensus motif (pT212, pT231, and pS235) or within this motif (pT217) would impact the competition for BIN1 SH3 domain binding. This phosphorylation corresponded to specific epitopes of Tau antibodies used to detect the pathological Tau protein, namely the AT180 epitope (pT231/pS235) (Goedert et al., 1994) and the AT100 epitope (pT212/pS214) (Zheng-Fischhofer et al., 1998).

pT231/pS235 residues affect the interaction by themselves, because full-length Tau phosphorylated on T231–S235, but not on T217, has a decreased affinity for BIN1 SH3 (Sottejeau et al., 2015). However, according to the structural model of BIN1 SH3/Tau (213–229) peptide (Figures 3, 4), T231-S235 residues did not make direct contact with BIN1 SH3 surface. Additional effects might thus be involved, and may be associated with the conformational modification of Tau induced by phosphorylation of T231 and S235 (Luna-Munoz et al., 2005; Sibille et al., 2012; Schwalbe et al., 2015). Alternatively, phosphorylation of T231 and S235 might act by a global electrostatic effect, partially neutralizing the K224-K225 basic motif that contacts the acidic BIN1 SH3 specificity region (Figure 4C). Substitution of K224 and K225 basic residues with neutral alanines indeed reduced the binding affinity by a factor of 10, confirming that K224 and/or K225 contribute significantly to the binding (Supplementary Figure S3). In addition, the structural model of BIN1 SH3/Tau (213–229) showed that T217 is accommodated by the proline-binding pocket of BIN1 SH3 (Figure 4). T217 phosphorylation in Tau (210–240) peptide may thus additionally weaken the interaction because of an unfavorable steric effect of the phosphate group.

Our results showed that conversely to Tau (210–240) peptide, phosphorylated Tau (210–240) peptide was not able to displace the intramolecular interaction of BIN1 SH3 with BIN1 CLAP domain, as its affinity for BIN1 SH3 was decreased compared to the unphosphorylated peptide. This confirmed our previous results that pT231 (together with other residues) weakens the BIN1/Tau interaction both *in vitro* and in primary neuronal cultures (Sottejeau et al., 2015). Here, a structure of the complex, based on NMR measurements, highlighted that T212, T231, and S235 did not make direct contact with BIN1 SH3 domain. T217, on the other hand, located in the bound proline-rich motif, but had however, its side-chain oriented in the opposite direction with respect to the domain surface (Figure 4). However, these combined phosphorylations decreased the affinity of BIN1 SH3 for the Tau peptide, either from their global electrostatic contribution, or by their impact on the peptide structure (Luna-Munoz et al., 2005; Sibille et al., 2012; Schwalbe et al., 2015).

Addition of Tau excess to BIN1 isoform 1 disfavors the intramolecular interaction whereas it favors an intermolecular interaction with a Tau proline-rich motif (Malki et al., 2017). Conversely, phosphorylation of Tau decreased the BIN1-SH3 domain’s affinity for Tau and pushed the dynamic equilibrium toward the intramolecular interaction, and might thus indirectly influence the availability of BIN1-CLAP domain. The proline-directed sites within Tau (210–240) might thus be the target of a transduction pathway; the activation of Pro-directed kinases would result in a decrease of BIN1’s affinity for Tau and (for BIN1 isoform 1) modify the availability of the CLAP domain.

## Author Contributions

AL and IM performed the biochemical experiments. AL, IM, and F-XC performed the NMR experiments. AL solved the structure of the complex. EB participated in NMR data treatment and HM in protein sample preparation. AL, IM, and IL wrote the manuscript. J-CL and IL conceived the study.

## Conflict of Interest Statement

The authors declare that the research was conducted in the absence of any commercial or financial relationships that could be construed as a potential conflict of interest.

## Abbreviations

AD: Alzheimer’s disease
BIN1: bridging integrator-1
BIN1 SH3: SH3 domain of BIN1
HSQC: heteronuclear single quantum correlation tau
NMR: nuclear magnetic resonance
NOESY: nuclear overhauser effect spectroscopy
PRD: proline-rich domain
pS: phosphoserine
pT: phosphothreonine
RMSD: root mean square deviation
SH3: Scr-homology-3.
