# Supplementary material for: Structural Basis of Tau Interaction With BIN1 and Regulation by Tau Phosphorylation
Source: Front Mol Neurosci. 2018 Nov 14;11:421. doi: 10.3389/fnmol.2018.00421 (PMC6246682; doi:10.3389/fnmol.2018.00421)
Supplement: Supplementary file 1 [file Data_Sheet_1.PDF]

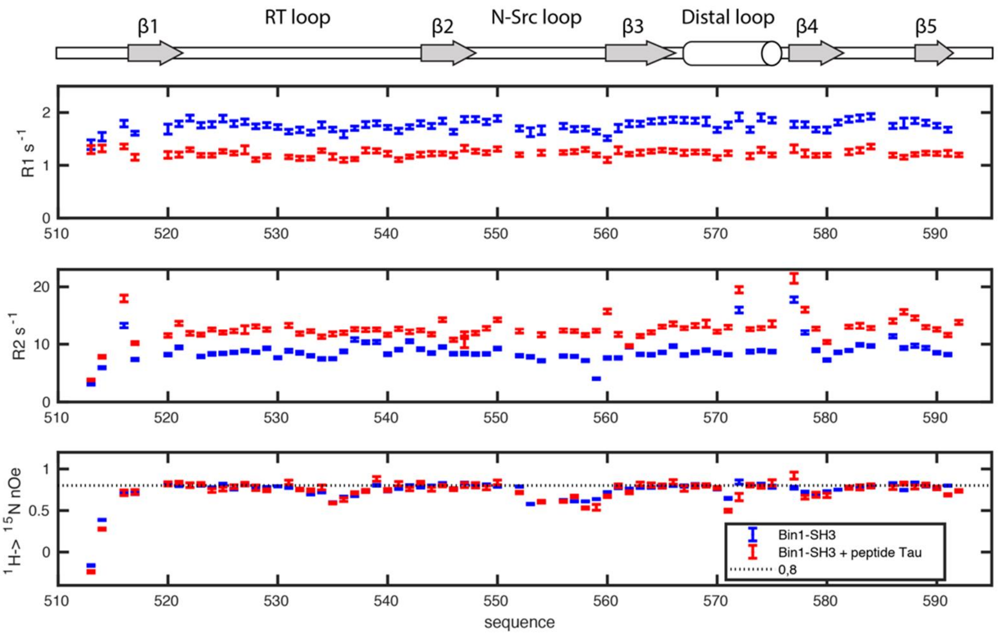

Fig. S1 Comparison of the dynamics of BIN1 SH3 and BIN1 SH3/Tau(210-240) complex. Free BIN1 SH3 in blue, BIN1 SH3 bound to Tau(210-240) in red; from top to bottom: R1 parameter, R2 parameter and residue-resolved $\{^1H\}$-^{15}N steady-state heteronuclear NOE. Secondary structure elements of BIN1 SH3 are indicated at the top.

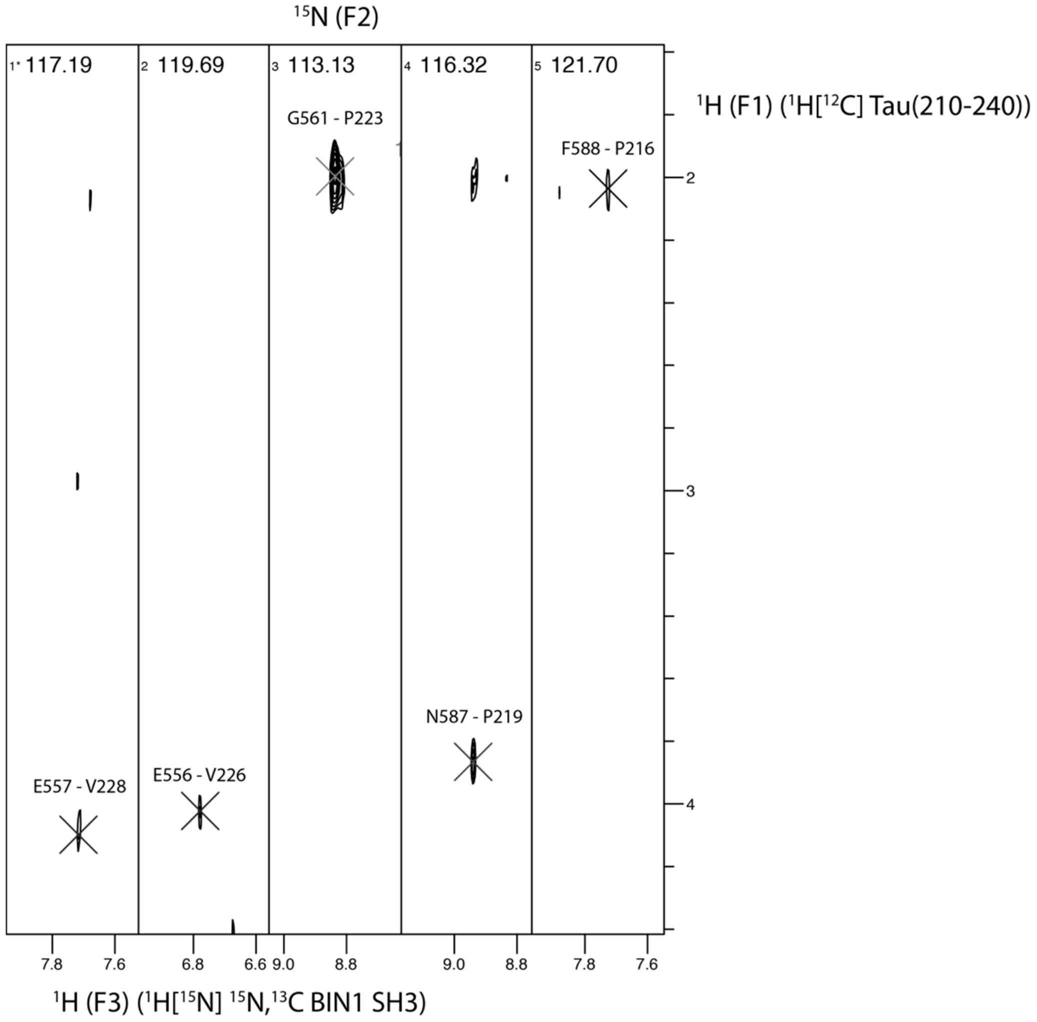

Fig. S2 Intermolecular NOEs in ^{1}H, ^{15}N 3D-NOESY-HSQC spectrum with $^{13}C/^{15}N$-filtering in F1 (selection of ^{1}H attached to ^{12}C and ^{14}N, Tau(210-240) signals), ^{15}N-editing in F2 and ^{1}H detection in F3 (^{1}H attached to ^{15}N, ^{15}N, ^{13}C-BIN1 SH3 signals), recorded at 950 MHz. NOEs are detected between protons of amide groups (^{1}H-^{15}N) of ^{15}N, ^{13}C BIN1 SH3 and protons (^{1}H-^{14}N and ^{1}H-^{12}C) of unlabelled Tau(210-240) peptide. Strips show the F1 dimension between 5 and 1 ppm, which corresponds to ^{1}H attached to carbon atoms in the side-chains of residues of Tau(210-240) peptide.

See Supplementary Table S3.

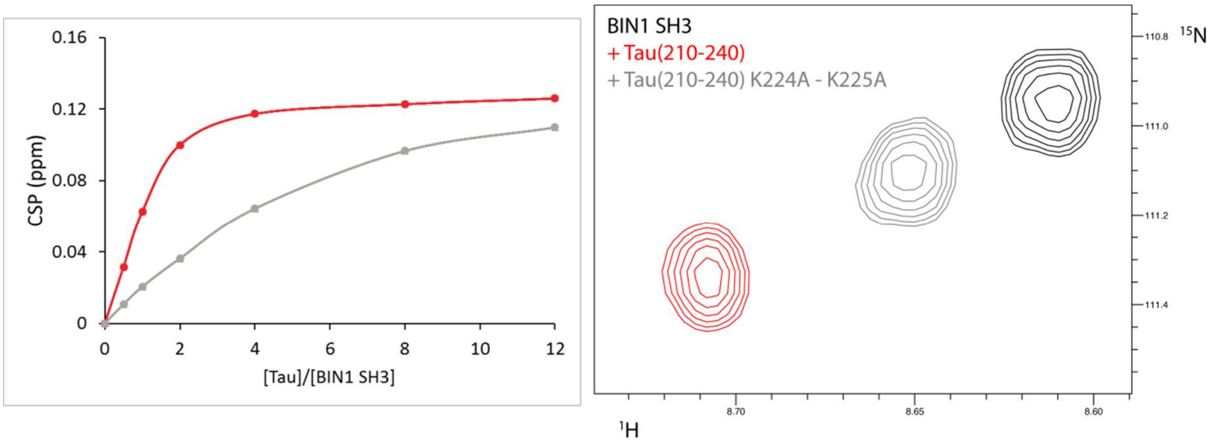

Fig S3 Binding affinity of KK-mutated Tau(210-240) peptide for ^{15}N-BIN1 SH3 - K224 was mutated to A224 and K225 to A225 in the peptide. At the right side, detail (showing resonance of T589) of overlaid ^{1}H-^{15}N HSQC spectra of BIN1 SH3 domain alone (in black) and in the presence of a 12-fold excess of Tau(210-240) peptide (in red), or of KK-mutated Tau(210-240) peptide (in gray). At the left side, saturation curves of BIN1 SH3 by Tau(210-240) peptide titration (in red) or KK-mutated Tau(210-240) peptide titration (in gray).

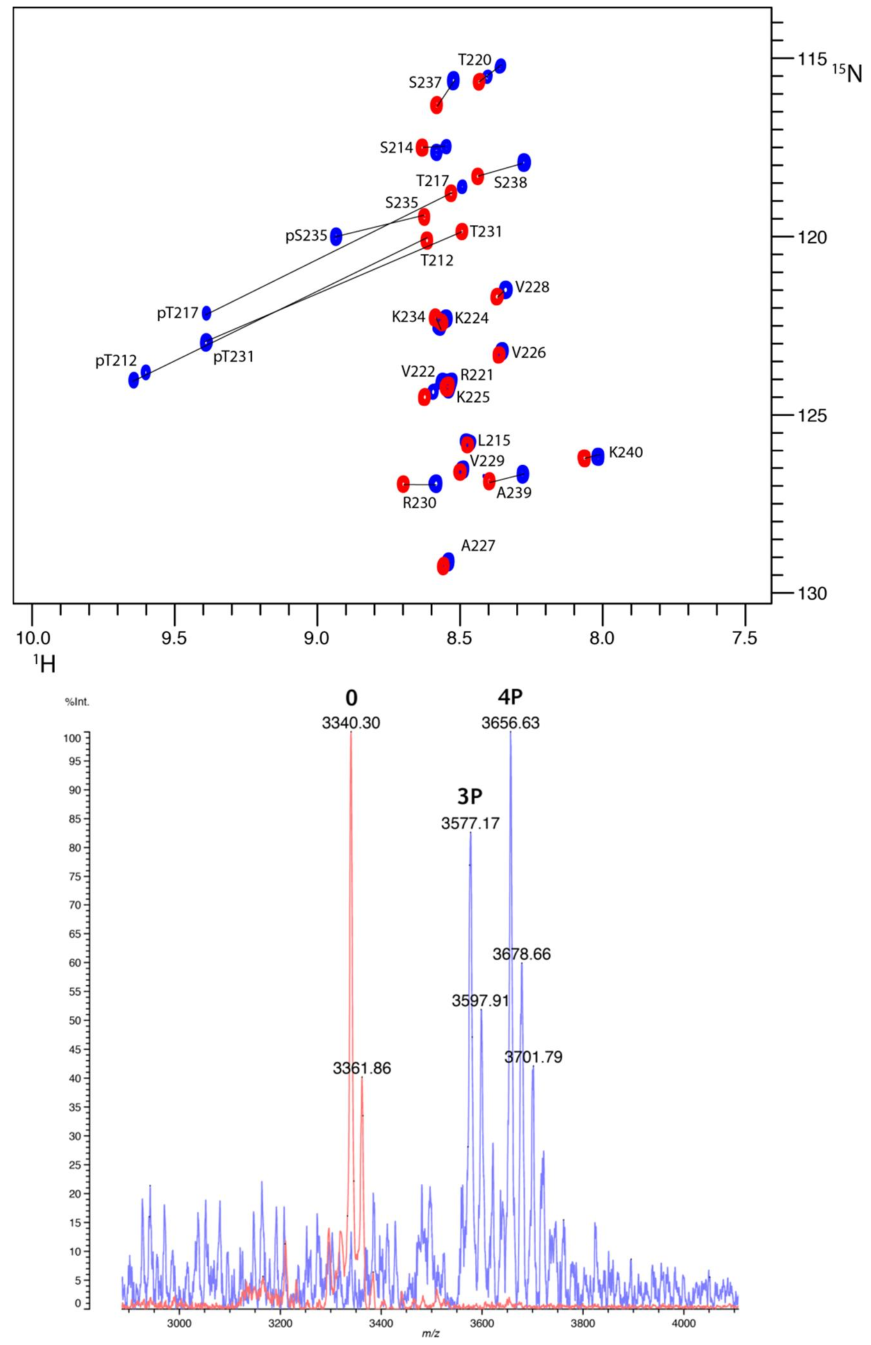

Fig. S4 Superimposed ^{1}H, ^{15}N HSQC spectra of Tau(210-240), in red, and of CDK2/CycA3 phosphorylated Tau(210-240), in blue. The resonances corresponding to the phosphorylated residues are labelled pS and pT. Two resonances are observed for pT212, corresponding to two populations, with four phosphorylated sites or three phosphorylated sites (without pT217). MALDI-TOF mass spectrometry analysis of ^{15}N-Tau(210-240) in red, and CDK2/CycA3 phosphorylated ^{15}N-Tau(210-240) in blue, showing 2 populations corresponding to 3 and 4 phosphorylation sites.

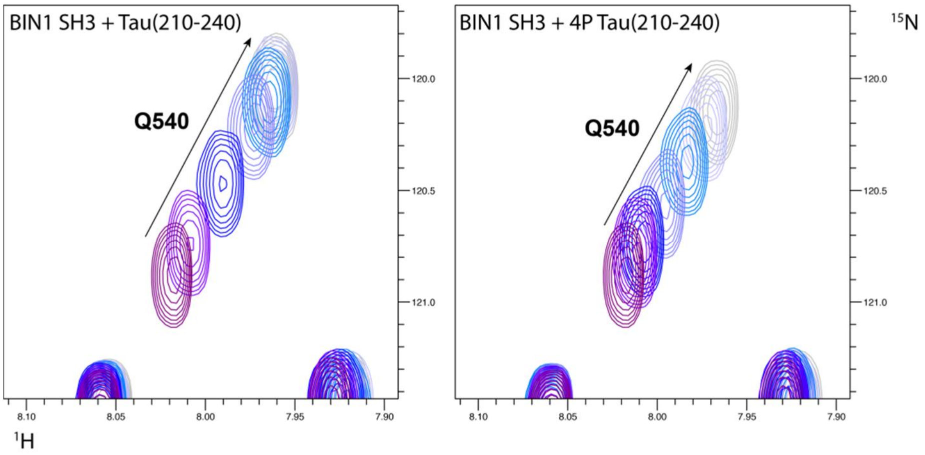

Fig. S5 Titration of ^{15}N-BIN1 SH3 with increasing concentration of Tau(210-240) peptide. Detail of overlaid ^{1}H-^{15}N HSQC spectra of BIN1 SH3 domain, in the presence of increasing amount of Tau(210-240) peptide, molar ratios peptide/BIN1 SH3 0, 0.5, 1, 2, 4, 8 and 12 (color scale from purple to gray), or of phospho-Tau(210-240) peptide, same molar ratios (same color scale). A spectrum is recorded for each titration point. The gradual change of the chemical shift value for each resonance (here resonance Q540), observed thanks to fast exchange between the free and bound forms of BIN1-SH3 domain, is next used to build a saturation curve. For Tau(210-240), resonances of peptide/BIN1 SH3 ratios 4, 8 and 12 are superimposed, indicating saturation. A larger excess of phospho-Tau(210-230) peptide is needed to reach saturation, due to its lower affinity for BIN1-SH3 domain, compared to the unphosphorylated peptide. Data corresponding to several resonances are averaged to estimate the Kd value.

Supplementary Table S1. ^{1}H, ^{15}N and ^{13}C chemical shift table for residues of Tau(210-240) peptide at 5°C. Columns are : Residue Type-Number, Atom, Nuclei, chemical shift values in ppm. (part per million)

S210	CA	13C	57.4
S210	CB	13C	63.5
S210	HA	1H	4.14
S210	HB2	1H	3.98
S210	HB3	1H	3.94
S210	N	15N	132.8
R211	C	13C	176.4
R211	CA	13C	56.2
R211	CB	13C	30.8
R211	CD	13C	43.2
R211	CG	13C	27.1
R211	HA	1H	4.44
R211	HB2	1H	1.74
R211	HB3	1H	1.80
R211	N	15N	122.6
R211	QD	1H	3.19
R211	QG	1H	1.63
T212	CA	13C	60.1
T212	CB	13C	69.6
T212	CG2	13C	20.9
T212	HN	1H	8.59
T212	HA	1H	4.57
T212	HB	1H	4.11
T212	HG1	1H	1.26
T212	N	15N	120.0
P213	C	13C	176.8
P213	CA	13C	63.2
P213	CB	13C	32.1
P213	CD	13C	51.1
P213	CG	13C	27.5
P213	HA	1H	4.44
P213	HB2	1H	2.32
P213	HB3	1H	1.90
P213	HD2	1H	3.73
P213	HD3	1H	3.90
P213	N	15N	139.7
P213	QG	1H	2.02
S214	C	13C	174.2
S214	CA	13C	58.3
S214	CB	13C	63.8
S214	HN	1H	8.61
S214	HA	1H	4.42
S214	N	15N	117.4
S214	QB	1H	3.84
L215	CA	13C	53.0
L215	CB	13C	41.7
L215	CG	13C	27.1
L215	HN	1H	8.46
L215	HA	1H	4.65
L215	HG	1H	1.59
L215	N	15N	125.8
L215	QB	1H	1.58
L215	QQD	1H	0.94
L215	CD*	13C	24.14
P216	C	13C	176.9
P216	CA	13C	62.8
P216	CB	13C	32.1
P216	CD	13C	50.6

P216	CG	13C	27.4
P216	HA	1H	4.47
P216	HB2	1H	2.30
P216	HB3	1H	1.89
P216	HD2	1H	3.65
P216	HD3	1H	3.85
P216	N	15N	136.4
P216	QG	1H	2.01
T217	CA	13C	60.1
T217	CB	13C	69.7
T217	CG2	13C	20.8
T217	HN	1H	8.51
T217	HA	1H	4.52
T217	HB	1H	4.08
T217	HG1	1H	1.26
T217	N	15N	118.7
P218	CA	13C	61.6
P218	CB	13C	30.8
P218	CD	13C	51.1
P218	CG	13C	27.4
P218	HA	1H	4.70
P218	HB2	1H	1.88
P218	HB3	1H	2.38
P218	HD2	1H	3.93
P218	HD3	1H	3.69
P218	HG2	1H	2.01
P218	HG3	1H	2.05
P218	N	15N	141.3
P219	C	13C	177.1
P219	CA	13C	62.9
P219	CB	13C	32.1
P219	CD	13C	50.6
P219	CG	13C	27.4
P219	HA	1H	4.50
P219	HB2	1H	2.32
P219	HB3	1H	1.91
P219	HD2	1H	3.83
P219	HD3	1H	3.67
P219	N	15N	135.8
P219	QG	1H	2.02
T220	C	13C	176.7
T220	CA	13C	62.0
T220	CB	13C	69.8
T220	CG2	13C	21.1
T220	HN	1H	8.42
T220	HA	1H	4.27
T220	HB	1H	4.19
T220	N	15N	115.6
T220	QG2	1H	1.21
R221	C	13C	176.0
R221	CA	13C	55.7
R221	CB	13C	30.8
R221	CD	13C	43.3
R221	CG	13C	27.1
R221	HN	1H	8.54
R221	HA	1H	4.37
R221	HB2	1H	1.81
R221	HB3	1H	1.74
R221	N	15N	124.2
R221	QD	1H	3.19
R221	QG	1H	1.62
E222	CA	13C	54.3

E222	CB	13C	29.6
E222	CG	13C	35.8
E222	HN	1H	8.61
E222	HA	1H	4.56
E222	HB2	1H	1.89
E222	HB3	1H	2.01
E222	N	15N	124.4
E222	QG	1H	2.29
P223	C	13C	176.8
P223	CA	13C	62.9
P223	CB	13C	32.1
P223	CD	13C	50.8
P223	CG	13C	27.5
P223	HA	1H	4.41
P223	HB2	1H	2.30
P223	HB3	1H	1.90
P223	HD2	1H	3.69
P223	HD3	1H	3.82
P223	N	15N	137.8
P223	QG	1H	2.02
K224	C	13C	176.7
K224	CA	13C	56.4
K224	CB	13C	32.9
K224	CD	13C	29.2
K224	CE	13C	42.1
K224	CG	13C	24.8
K224	HN	1H	8.55
K224	HA	1H	4.23
K224	HG2	1H	1.45
K224	HG3	1H	1.45
K224	N	15N	122.3
K224	QB	1H	1.77
K224	QD	1H	1.68
K224	QE	1H	2.99
K225	C	13C	176.5
K225	CA	13C	56.2
K225	CB	13C	33.0
K225	CD	13C	29.1
K225	CE	13C	42.4
K225	CG	13C	24.7
K225	HN	1H	8.54
K225	HA	1H	4.32
K225	N	15N	124.2
K225	QB	1H	1.77
K225	QD	1H	1.69
K225	QE	1H	3.19
K225	QG	1H	1.43
V226	C	13C	175.8
V226	CA	13C	62.0
V226	CB	13C	32.9
V226	HN	1H	8.35
V226	HA	1H	4.07
V226	HB	1H	2.01
V226	N	15N	123.3
V226	QQG	1H	0.93
V226	CG*	13C	21.0
A227	C	13C	177.5
A227	CA	13C	52.3
A227	CB	13C	19.1
A227	HN	1H	8.55
A227	HA	1H	4.33
A227	N	15N	129.2

A227	QB	1H	1.36
V228	C	13C	176.2
V228	CA	13C	62.3
V228	CB	13C	32.9
V228	HN	1H	8.36
V228	HA	1H	4.04
V228	HB	1H	2.00
V228	N	15N	121.6
V228	QQG	1H	0.93
V228	CG*	13C	21.0
V229	C	13C	176.0
V229	CA	13C	62.2
V229	CB	13C	32.7
V229	HN	1H	8.49
V229	HA	1H	4.08
V229	HB	1H	2.01
V229	N	15N	126.6
V229	QQG	1H	0.93
V229	CG*	13C	20.9
R230	C	13C	176.2
R230	CA	13C	55.7
R230	CB	13C	30.8
R230	CD	13C	43.2
R230	CG	13C	27.1
R230	HN	1H	8.69
R230	HA	1H	4.41
R230	HB2	1H	1.79
R230	HB3	1H	1.75
R230	N	15N	126.9
R230	QD	1H	3.18
R230	QG	1H	1.63
T231	CA	13C	59.9
T231	CB	13C	69.6
T231	CG2	13C	20.9
T231	HN	1H	8.47
T231	HA	1H	4.55
T231	HB	1H	4.09
T231	N	15N	119.8
T231	QG2	1H	1.25
P232	CA	13C	61.6
P232	CB	13C	30.9
P232	CD	13C	51.2
P232	CG	13C	27.5
P232	HA	1H	4.68
P232	HB2	1H	2.13
P232	HB3	1H	2.38
P232	HD2	1H	3.90
P232	HD3	1H	3.69
P232	HG2	1H	2.01
P232	HG3	1H	2.05
P232	N	15N	140.9
P233	C	13C	176.8
P233	CA	13C	62.7
P233	CB	13C	32.1
P233	CD	13C	50.6
P233	CG	13C	27.5
P233	HA	1H	4.43
P233	HB2	1H	2.30
P233	HB3	1H	1.90
P233	HD2	1H	3.81
P233	HD3	1H	3.64
P233	N	15N	135.9

P233	QG	1H	2.02
K234	C	13C	176.7
K234	CA	13C	56.2
K234	CB	13C	33.1
K234	CD	13C	29.2
K234	CE	13C	42.1
K234	CG	13C	24.7
K234	HN	1H	8.58
K234	HA	1H	4.28
K234	N	15N	122.2
K234	QB	1H	1.77
K234	QD	1H	1.68
K234	QE	1H	2.99
K234	QG	1H	1.45
S235	CA	13C	56.5
S235	CB	13C	63.2
S235	HN	1H	8.61
S235	HA	1H	4.75
S235	N	15N	119.4
S235	QB	1H	3.86
P236	C	13C	177.1
P236	CA	13C	63.4
P236	CB	13C	32.1
P236	CD	13C	50.9
P236	CG	13C	27.4
P236	HA	1H	4.48
P236	HB2	1H	2.32
P236	HB3	1H	1.90
P236	HD2	1H	3.75
P236	HD3	1H	3.85
P236	N	15N	138.3
P236	QG	1H	2.03
S237	C	13C	174.7
S237	CA	13C	58.3
S237	CB	13C	63.8
S237	HN	1H	8.56
S237	HA	1H	4.46
S237	N	15N	116.2
S237	QB	1H	3.89
S238	C	13C	174.1
S238	CA	13C	58.4
S238	CB	13C	63.8
S238	HN	1H	8.42
S238	HA	1H	4.46
S238	N	15N	118.3
S238	QB	1H	3.88
A239	C	13C	176.8
A239	CA	13C	52.6
A239	CB	13C	19.2
A239	HN	1H	8.39
A239	HA	1H	4.34
A239	N	15N	126.9
A239	QB	1H	1.39
K240	CA	13C	57.7
K240	CB	13C	33.6
K240	CD	13C	29.2
K240	CE	13C	42.2
K240	CG	13C	24.8
K240	HN	1H	8.05
K240	HA	1H	4.12
K240	N	15N	126.2
K240	QB	1H	1.81

K240	QD	1H	1.67
K240	QE	1H	2.99
K240	QG	1H	1.40

Supplementary Table S2. ^{1}H, ^{15}N and ^{13}C chemical shift table for residues of phosphorylated Tau(210-240) peptide at 5°C. Values are in ppm. (part per million)

Residue	AA	HA	CA	CB	CO	N	HN
210	S	4.15	57.6	63.1	0.0	0.0	0.00
211	R	4.27	56.3	29.9	0.0	120.7	0.00
212	T	4.42	61.3	72.4	176.0	123.7	9.59
213	P	4.45	63.3	32.1	176.8	141.9	0.00
214	S	4.41	58.3	64.0	174.2	117.5	8.55
215	L	4.66	0.0	0.0	0.0	125.8	8.47
216	P	4.47	62.8	32.1	175.7	136.4	0.00
217	T	4.43	60.8	72.7	0.0	121.9	9.35
218	P	4.71	61.7	30.9	0.0	142.9	0.00
219	P	4.50	62.9	32.1	177.1	135.9	0.00
220	T	4.28	61.9	69.8	176.7	115.2	8.36
221	R	4.36	55.7	31.0	176.0	124.1	8.53
222	E	4.56	54.3	29.6	0.0	124.0	8.56
223	P	4.40	62.9	32.1	176.8	137.8	0.00
224	K	4.22	56.3	32.9	176.7	122.3	8.55
225	K	4.32	56.1	32.9	176.5	124.3	8.54
226	V	4.06	62.0	32.8	175.7	123.2	8.35
227	A	4.32	52.2	19.1	177.5	129.1	8.54
228	V	4.04	62.3	32.8	176.1	121.5	8.34
229	V	4.09	62.1	32.8	175.9	126.5	8.49
230	R	4.36	55.7	30.9	175.7	126.9	8.58
231	T	4.47	60.7	72.7	0.0	122.8	9.36
232	P	4.67	61.7	30.9	0.0	142.5	0.00
233	P	4.43	62.7	32.2	176.8	136.1	0.00
234	K	4.31	55.9	33.2	176.6	122.5	8.57
235	S	4.79	55.6	65.0	0.0	120.0	8.93
236	P	4.44	63.9	32.0	177.5	138.4	0.00
237	S	4.41	59.0	63.1	175.0	115.6	8.52
238	S	4.46	58.5	63.9	174.1	117.9	8.28
239	A	4.33	52.6	19.1	176.8	126.7	8.28
240	K	4.13	57.6	33.6	0.0	126.2	8.01

Supplementary Table S3. List of intermolecular NOE contacts between $^{1}H(^{15}N)$-BIN1 SH3 (2D F1-F2) and ^{1}H-Tau(210-240) peptide (F3) in the complex. See also Supplementary Figure S2.

	Position F1	Position F2	Position F3	Assign F1	Assign F2	Assign F3
1	7.71642	117.2033	4.09844	557GluH	557GluN	(228Val/229Val)Ha
2	6.7782	119.6777	4.02268	558GlnH	558GlnN	226ValHa
3	8.83643	113.1811	1.99649	561GlyH	561GlyN	223ProHgb
4	8.93987	116.3431	3.86601	587AsnH	587AsnN	219ProHdb
5	7.72578	121.7052	2.03527	588PheH	588PheN	216ProHgb

Supplementary Table S4. Statistics related to the calculation by HADDOCK of the water-refined model structures of BIN1 SH3 with Tau(210-240). The reported statistics correspond to the average of the best four structures within the cluster. The most populated, Cluster 1 had better statistics and was chosen as representative of the complex.

	**Cluster 1**	**Cluster 2**
HADDOCK score (a.u.)	-101.6 +/- 0.8	-58.5 +/- 4.7
Cluster size	190	6
RMSD from the overall lowest-energy structure (Å)	0.6 +/- 0.5	1.6 +/- 0.1
Van der Waals energy (kcal/mol)	-37.1 +/- 3.8	-21.3 +/- 5.4
Electrostatic energy (kcal/mol)	-348.3 +/- 26.6	-229.8 +/- 43.0
Desolvation energy (kcal/mol)	4.3 +/- 3.6	7.8 +/- 3.6
Restraints violation energy (kcal/mol)	8.2 +/- 2.04	10.3 +/- 2.64
Buried Surface Area ($Å^2$)	1174.4 +/- 72.7	925.4 +/- 69.9
